# Choosing proper fluorescent dyes, proteins, and imaging techniques to study mitochondrial dynamics in mammalian cells

**DOI:** 10.1007/s41048-017-0037-8

**Published:** 2017-03-24

**Authors:** Xingguo Liu, Liang Yang, Qi Long, David Weaver, György Hajnóczky

**Affiliations:** 1 Key Laboratory of Regenerative Biology, Guangdong Provincial Key Laboratory of Stem Cell and Regenerative Medicine, South China Institute for Stem Cell Biology and Regenerative Medicine, Guangzhou Institutes of Biomedicine and Health, Chinese Academy of Sciences, Guangzhou, 510530 China; 2 Department of Pathology, MitoCare Center, Anatomy and Cell Biology, Thomas Jefferson University, Philadelphia, PA 19107 USA

**Keywords:** Imaging, Photoactivation, Photoswitching, Mitochondrial dynamics, Super-resolution microscope

## Abstract

Mitochondrial dynamics refers to the processes maintaining mitochondrial homeostasis, including mitochondrial fission, fusion, transport, biogenesis, and mitophagy. Mitochondrial dynamics is essential for maintaining the metabolic function of mitochondria as well as their regulatory roles in cell signaling. In this review, we summarize the recently developed imaging techniques for studying mitochondrial dynamics including: mitochondrial-targeted fluorescent proteins and dyes, live-cell imaging using photoactivation, photoswitching and cell fusion, mitochondrial transcription and replication imaging by in situ hybridization, and imaging mitochondrial dynamics by super-resolution microscopy. Moreover, we discuss examples of how to choose and combine proper fluorescent dyes and/or proteins.

## Introduction

The dynamic properties of mitochondria (mitochondrial fission, fusion, transport, biogenesis, and degradation) are critical for maintaining electron transfer chain function and electrical connectivity of mitochondria, preventing build-up of damaged proteins, protecting mitochondrial DNA (mtDNA) integrity, controlling mitochondrial turnover, and regulating many signaling pathways (McBride *et al*. 2006; Detmer and Chan 2007; Tatsuta and Langer 2008; Liu *et al*. 2009; Youle and van der Bliek 2012; Liesa and Shirihai 2013; Friedman and Nunnari 2014). Mitochondria continually exchange contents including proteins, mitochondrial RNA (mtRNA), and mtDNA through mitochondrial fusion. Mitochondrial fusion–fission dynamics, transport, and biogenesis/mitophagy also determine mitochondrial morphology, position, and amount. Mitochondria have distinctive features including their own genome, two layers of membranes, and the existence of inner membrane potential, which determine the unique features of mitochondrial dynamics including sequential fusion of two layers of mitochondrial membranes and selective fusion controlled by mitochondrial membrane potential. These features make mitochondrial imaging different from many other organelles.

Fluorescent sensors have attracted considerable attention in mitochondrial dynamics as they enable real-time monitoring and imaging of sub-cellular structures. Improvements in microscopy and the development of mitochondria-specific imaging techniques have greatly advanced the study of mitochondrial dynamics. For example, fluorescence recovery after photobleaching (FRAP) studies have been developed to understand mitochondrial dynamics (Mitra and Lippincott-Schwartz 2010). Several studies have investigated mitochondrial dynamics in plants (Logan and Leaver 2000; Matsushima *et al*. 2008; Ekanayake *et al*. 2015); here, we will highlight the ongoing research into mitochondrial dynamics in mammalian cells. We summarize the recently developed imaging techniques and discuss examples of how to properly choose and combine fluorescent dyes and/or proteins.

## Mitochondria-Specific Fluorescent Proteins and Dyes

Mitochondrial-targeted fluorescent proteins (FPs) have been widely used. Specific localization is achieved by fusing the FP gene to the mitochondrial targeting sequence of an endogenous protein. The targeting sequence of cytochrome *c* oxidase subunit VIII, which localizes in the mitochondrial matrix, is the most widely used one. According the demands of experiments, one can choose different mitochondrial targeting sequences using software prediction algorithms (Fukasawa *et al*. 2015; Martelli *et al*. 2015). Several effective targeting sequences have also been found for the outer surface of the outer membrane, based on AKAP1, OMP25, and TOM70(Huang *et al*. 1999; Suzuki *et al*. 2002; Malka *et al*. 2005), and the outer surface of the inner membrane using the Cox8a subunit of complex IV(Wilkens *et al*. 2013).

Fluorescent dyes have also been used to mark mitochondria. Several fluorescent probes including Rhodamine 123 (R123), tetramethylrhodamine methyl ester (TMRM), and JC-1 can be used to monitor mitochondrial membrane potential ($\Delta \varPsi {}_{\text{m}}$) in various cell types (Joshi and Bakowska 2011), making them suitable for marking functional mitochondria. TMRM did not suppress respiration when it was used at low concentrations, making it a good and popular choice for marking functional mitochondria (Scaduto and Grotyohann 1999). The membrane-permeant JC-1 dye exhibits potential-dependent accumulation in mitochondria, indicated by a fluorescence emission shift from green (~529 nm) to red (~590 nm). Due to the spectral overlap with red- and green-fluorescent dyes and proteins, it is difficult to use JC-1 in a multi-parameter measurement. TMRM and mtGFP can be combined to study high and low $\Delta \varPsi {}_{\text{m}}$ of mitochondria in cells under normal and stress conditions (Twig *et al*. 2008). However, these $\Delta \varPsi {}_{\text{m}}$ sensors are not optimal for marking mitochondrial morphology since they are easily washed out of cells once the mitochondria experience a loss in $\Delta \varPsi {}_{\text{m}}$. They are also unsuitable for experiments that require cells to be treated with aldehyde fixatives or with other agents that affect the metabolic state of the mitochondria. MitoTracker^®^ probes including MitoTracker Green and MitoTracker Red are fixable probes that are not dependent on $\Delta \varPsi {}_{\text{m}}$ (Chazotte 2011). Although they cannot be used to study $\Delta \varPsi {}_{\text{m}}$, they are good markers for mitochondrial morphology.

To overcome some of the deficiencies in the above-mentioned stains, including poor photostability and cytotoxicity, new alternatives have recently been developed, including NPA-TPP (Huang *et al*. 2015b) and Splendor (Carvalho *et al*. 2014), which not only have high specificity to mitochondria, but also exhibit high photostability, negligible cytotoxicity, good water solubility, better fluorescence intensity (FI), and chemical stability in living cells. The extraordinarily high photostability of NPA-TPP is attributed to its extremely stable structure owing to the high π-conjugated macrocycle and strong fluorescent response (Huang *et al*. 2015a), and Splendor is selected from a series of new rationally designed 2,1,3-benzothiadiazole (BTD) fluorescent derivatives with excellent fluorescence intensity and almost no background signal (Carvalho *et al*. 2014). Another probe, named MitoBADY, can be also used to visualize mitochondria in living cells by Raman microscopy (Yamakoshi *et al*. 2015). These stains showed great potential for marking mitochondria and enable real-time and long-term tracking mitochondrial dynamics. The properties and applications of these dyes are summarized in Table 1.

**Table 1:** The properties and applications of mitochondria-specific dyes

Fluorescent dyes	Properties	Applications
Rhodamine 123	Negligible cytotoxicity and potential-sensitive	Measure $\Delta \varPsi {}_{\text{m}}$ and mark functional mitochondria
JC-1	Dual-emission and potential-sensitive	Measure $\Delta \varPsi {}_{\text{m}}$
TMRM/TMRE	Potential-sensitive	Measure $\Delta \varPsi {}_{\text{m}}$, mark functional mitochondria and image time-dependent $\Delta \varPsi {}_{\text{m}}$
MitoTracker^®^ probes	Fixable	Mark mitochondria
NPA-TPP	High photostability, negligible cytotoxicity, and good water solubility	Long-term tracking
Splendor	Excellent fluorescence intensity and negligible background signal	Long-term tracking
MitoBADY	High sensitivity and specificity	Long-term tracking

## Visualizing Mitochondrial Dynamics by Live-Cell Imaging

### Photoactivation

**Fig. 1:**
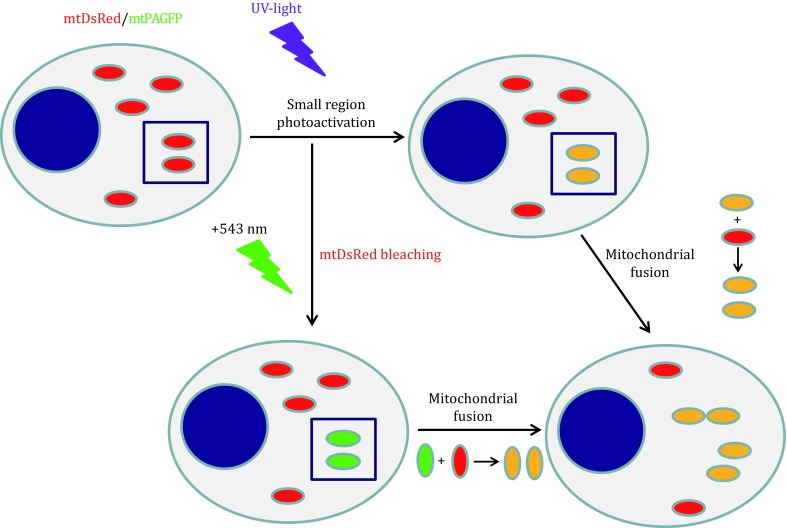
The combination of mtPAGFP photoactivation with mtDsRed bleaching. PAGPF can be photoactivated by UV-light from a non-fluorescent to a green-fluorescent state. Mitochondria expressing mtPAGFP and mtDsRed are marked in *red* and the nucleus is marked in *blue* in this figure. After small region photoactivation, mitochondria in the photoactivated region are labeled as *yellow* due to the overlapping of *red* and *green*. The combination of mtPAGFP photoactivation with mtDsRed bleaching by 543 nm laser can mark the photoactivated mitochondria with single green fluorescence. When the non-photoactivated mitochondria labeled as *red* fuse with *yellow* mitochondria or *green* mitochondria, the color of both mitochondria become *yellow* for the acquirement of GFP fluorescence

Photoactivatable fluorescent proteins including PAGFP (Patterson and Lippincott-Schwartz 2002), Kindling fluorescent protein (KFP) (Chudakov *et al*. 2003), and photoactivatable mCherry (PAmCherry) (Subach *et al*. 2009) are widely used to visualize mitochondrial dynamics (Fig. 1). Using a microscope system with region-of-interest illumination capability, it is possible to mark a subset of mitochondria within a cell. Real-time imaging of photoactivatable fluorescent proteins has allowed the visualization of mitochondrial fusion by time-lapse imaging (Karbowski *et al*. 2014). Studies of the fusion dynamics of different mitochondrial sub-compartments, inner membrane (IMM), outer membrane (OMM), and inter-membrane space (IMS) have been achieved by fusing the photoactivatable FPs to the appropriate targeting signals or proteins. Single-molecule tracking in mitochondria was used to study mitochondrial dynamics including the diffusion or movement of mitochondrial membrane proteins. However, its application is limited (Kuzmenko *et al*. 2011; Appelhans *et al*. 2012). In most cases, a combination of one photoactivatable FP and an ordinary FP of another color is used to divide the mitochondria to two classes.

Photoactivatable FPs exhibit weak fluorescence before photoactivation and strong fluorescence after photoactivation. Mitochondrial fusion can be determined by the transfer of photoactivated FP from a subset of mitochondria throughout the mitochondrial network. For instance, the cyan-photoactivated, green-fluorescent PAGFP (mtPAGFP) can be combined with mtRFP or mtDsRed to visualize the exchange of matrix contents between individual mitochondria in real time (Fig. 1). Using high-resolution confocal imaging with region-of-interest scanning, an irreversible photoactivation within the mitochondria is achieved by illumination with a pulse of UV-light (Patterson and Lippincott-Schwartz 2002). As the result of fusion, activated mtPAGFP molecules transfer to unlabeled mitochondria, also leading to a decrease in PAGFP FI of the mitochondria containing activated mtPAGFP. The combination of mitochondrial matrix-targeted, green-photoactivated, red-fluorescent KFP (mtKFP), and green or yellow fluorescent protein (mtGFP, mtYFP) may also be used to study mitochondrial dynamics (Liu *et al*. 2009), though the newer PAmCherry has faster maturation, better pH stability, faster photoactivation, higher photoactivation contrast, and better photostability (Subach *et al*. 2009).

The bleaching of one FP can be combined with the photoactivation of another to improve the visibility of fusion, for instance, using the combination described above of PAGFP and DsRed. Using two-photon excitation for photoactivation at ~750 nm effectively simultaneously bleaches DsRed and photoactivates PAGFP. Alternatively, high intensity from the green, DsRed excitation laser (typically 543 or 561 nm) can be used simultaneously with UV illumination to achieve the photoactivation/bleaching effect, which is illustrated in Fig. 1. This technique has helped show that transfer of matrix contents during mitochondrial fusion is un-directed, *i.e.* occurs by passive diffusion (Liu *et al*. 2009).

Combined photoactivation and bleaching is also used to study the transfer of mitochondrial proteins of the IMM, OMM, IMS, and matrix and of nucleoids between mitochondria, the targeted compartment can be marked with red fluorescence in combination with mtPAGFP, or alternatively PAGFP targeted to the sub-compartment of interest can be combined with mtDsRed. In the former case, mitochondrial fusion events can be recognized by the change of PAGFP FI, and the transfer of targeted proteins can be studied by observing the red fluorescence signal among the two mitochondria undergoing mitochondrial fusion. For instance, Tfam-DsRed marking mtDNA and mtPAGFP were combined to show the transfer of mtDNA nucleoids among mitochondria (Yang *et al*. 2015). Also, TMRM measurements of $\Delta \varPsi {}_{\text{m}}$ and mtPAGFP were combined to show that mitochondria with lower $\Delta \varPsi {}_{\text{m}}$ are less likely to undergo fusion (Twig *et al*. 2008).

The rate at which the activated mtPAGFP molecules equilibrate across the entire mitochondrial population is used as a measure of fusion activity. In most cases, measurements were performed using a single-cell time-lapse approach, quantifying the equilibration in one cell over a certain time period. Another approach is to use a programmable, motorized stage to alternate among fields and collect multiple cells simultaneously at lower temporal resolution for each cell. This provides a significant increase in the number of cells that can be acquired, but sacrifices the ability to resolve individual fusion events, while maintaining the highly resolved sub-cellular quantification (Lovy *et al*. 2012).

Photoactivatable fluorescent proteins have also been used to study the fate of mitochondria as they age in cells. First, mtPAGFP was expressed in stem cells. After exposure to a pulse of UV-light, mtPAGFP continued to be synthesized. Subsequently, cells accumulated unlabeled ‘young’ mitochondria which could be distinguished from the ‘old’ ones by different PAGFP FI. By following the fates of old and young organelles during the division of human mammary stem-like cells, it was shown that cells apportion the mitochondria asymmetrically between daughter cells according to age (Katajisto *et al*. 2015).

### Photoswitching

**Fig. 2:**
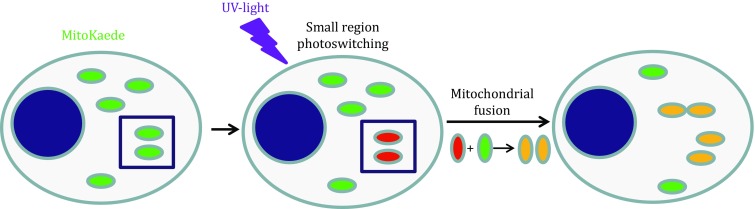
Photoswitching of MitoKaede. Mitochondria expressing MitoKaede are marked in *green*, and nucleus is marked in *blue* in this figure. After small region of photoswitching with UV-light, mitochondria change color from *green* to *red*. When photoswitched mitochondria labeled as *red* fuse with mitochondria labeled as *green*, the color of mitochondria become *yellow* for the acquirement of GFP fluorescence

Photoswitching fluorescent proteins, which change color upon selective illumination, including Kaede (Ando *et al*. 2002; Owens and Edelman 2015), Dendra2 (Pham *et al*. 2012) and Eos (Wiedenmann *et al*. 2004) have also been used to visualize mitochondrial dynamics. Similar to the experiments described for monitoring mitochondrial fusion by photoactivation, a subset of mitochondria may be irreversibly photoswitched from green to red emitting by targeted illumination of labeled organelles; however, expression of only one protein is required. These color-changing properties provide a simple and powerful technique for regional optical marking (Ando *et al*. 2002). Mitochondrial-targeted Kaede (MitoKaede) has been used to study mitochondrial dynamics (Owens and Edelman 2015), in the manner illustrated in Fig. 2. MitoKaede was also used to observe mitochondrial dynamics within neuronal processes to show that there is a widespread exchange of mitochondrial components throughout a neuron as a result of organelle fusion (Owens and Walcott 2012). A mouse line expressing mitochondrial Dendra2 (mito-Dendra2) has been generated to study mitochondrial dynamics in primary tissue (Pham *et al*. 2012).

### Mitochondrial fusion assayed by cell fusion

**Fig. 3:**
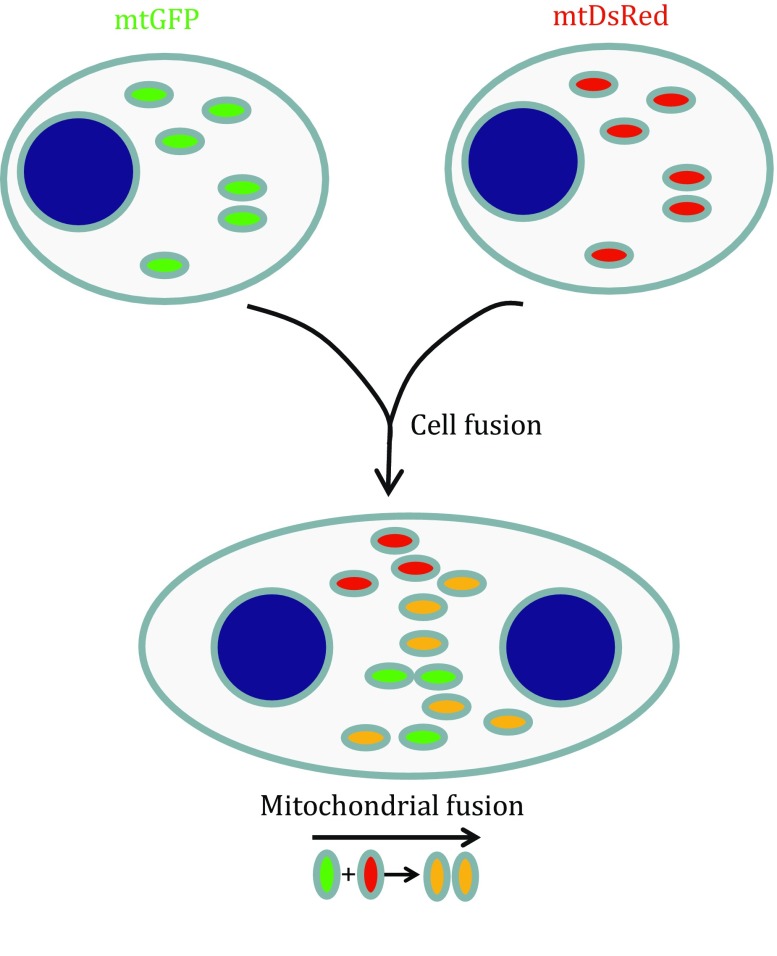
Cell fusion model using cells expressing mtDsRed and cells expressing mtGFP. Mitochondria expressing mtGFP or mtDsRed are marked in *green* or *red*, and nucleus is marked in *blue* in this figure. In cell hybrids between cells expressing mtGFP and cells mtDsRed, mitochondria become *yellow* after *green*-labeled mitochondria fuse with *red*-labeled mitochondria

Cell fusion by treatment with polyethylene glycol (PEG) provides another way to study mitochondrial fusion dynamics. As illustrated in Fig. 3, a cell fusion model is generated using cells with mitochondria labeled in two different colors. Practically, cells expressing mtGFP and others with mtDsRed are mixed and plated commingled. The cells are kept in the presence of cycloheximide from before PEG treatment to block any de novo FP synthesis. Fused cells were imaged after 7 h to visualize the dramatic loss of fusion activity from deletion of mitofusin proteins (Chen *et al*. 2003; Liu *et al*. 2009), but to visualize fusion events in normal cells, imaging should be done within 2 h (Liu *et al.* 2009).

## Mitochondrial Transcription and Replication Imaging by In Situ Hybridization

Understanding the replication and transcription of mtDNA is important to a complete picture of mitochondrial dynamics. The mitochondrial transcription and replication imaging protocol (mTRIP) is a modified fluorescence in situ hybridization approach that specifically targets replicating mtDNA nucleoids and can simultaneously be used to exhibit transcription activity by targeting mtRNA. When combined with labeling of the mitochondria by immunofluorescence or MitoTracker loading, mTRIP allows classification of the organelles by replication and/or transcription activity, which has shown to change depending on physiological conditions (Chatre and Ricchetti 2015).

## Imaging Mitochondrial Dynamics by Super-Resolution Microscopy

Conventional confocal microscopy techniques have a limited resolving power of ~250 nm, due to the properties of light. This diffraction limit is close to the size of mitochondria, which are typically 0.5–1 μm in diameter. However, newly developed super-resolution imaging technologies supply new ways to analyze mitochondrial dynamics and the interactions among sub-cellular organelles. The following four methods of super-resolution microscopy have been commercialized: SIM (Gustafsson 2005), STED (Hell 2003), PALM(Betzig *et al*. 2006)/STORM (Rust *et al*. 2006), and Zeiss Airyscan. Any ordinary fluorescent probes and dyes are in principle usable in SIM and Airyscan imaging. Both of these techniques provide resolution of ~130 nm in commercial devices, making it possible to visualize that mitochondria contain different compartments, *i.e.,* distinguish outer membrane from matrix (Gustafsson 2000, 2005). However, with these techniques, it is still not possible to visualize the dynamics of the two membranes of mitochondria, including the events of protein interactions and membrane detaching (Kukat *et al*. 2011; Jans *et al*. 2013). STED and PALM technologies can attain better resolution, 50 and 20 nm, respectively (Rust *et al*. 2006; Shim *et al*. 2012; Jans *et al*. 2013). However, not all fluorescent dyes targeted to mitochondria could be used in these two methods and combinations of multiple colors remain especially difficult. However, PALM imaging of mitochondrial dynamics in live cells using a single color has been achieved (Fernandez-Suarez and Ting 2008; Shim *et al*. 2012; Shcherbakova *et al*. 2014).

Fluorophores applied for PALM imaging of mitochondrial dynamics must have appropriate ability to switch between dark and fluorescing states, and should have a high quantum efficiency to minimize photodamage of the sample (Patterson *et al*. 2010; Shcherbakova *et al*. 2014). Three fluorescent dyes have been used in PALM imaging of mitochondrial dynamics: MitoTracker Orange, MitoTracker Red, and MitoTracker Deep Red (Shim *et al*. 2012). The cells are labeled according to the ordinary protocol, but should be imaged with PALM system with an extremely high-speed camera (such as sCMOS) to avoid artifacts from the high speed of diffusion of the dyes. The SNAP-tag and CLIP-tag (NEB, MA) or HaloTag (Promega) systems could also be used in super-resolution imaging of mitochondrial dynamics (Klein *et al*. 2011; Lukinavicius *et al*. 2013). Mitochondria-targeted proteins carrying these tags can be imaged with PALM after conjugation with proper fluorescence dyes, such as TMR-Star (Klein *et al*. 2011) and SIR (Lukinavicius *et al*. 2013). Multi-color super-resolution images of mitochondrial dynamic events can also be achieved in this way.

There are three kinds of FPs that could be applicable in super-resolution imaging, including photoactivatable FPs (PAFPs), photoswitchable FPs (PSFPs), and reversely photoswitchable FPs (rsFPs) (Shcherbakova *et al*. 2014). PAFPs are not ideal for single-color imaging with PALM as these FPs are invisible in pre-imaging, making it difficult to find the interesting areas and events. They are, however, in principle useful for multi-color super-resolution imaging since each occupies only one color. Both PAGFP and PAmCherry can be used in PALM imaging, though their optical properties are far from optimal.

Many PSFPs have already been used in time-lapse imaging of mitochondrial dynamics as described above, including mEOS family proteins, mKiKGR, and Dendra2. Each of these could also in principle be used in PALM imaging. PSmOrange and PSmOrange2 have potential for use in multi-color PALM imaging using the far-red spectrum (Subach *et al*. 2011).

rsFPs can be switched on or off by an illumination at different wavelengths. They are potentially useful for studying mitochondrial dynamics with PALM. However, there are few rsFPs that could be applicable in PALM imaging due to the requirement for high quantum efficiency and long half-time period. Dronpa family members such as Dronpa2 and Dronpa3 have been successfully applied in PALM imaging of mitochondria (Rocha *et al*. 2014). Newly developed mGeos-M is derived from mEos2 and was reported to also be effective in PALM imaging (Chang *et al*. 2012). Thus, it is another candidate for mitochondrial dynamics with PALM imaging.

## Discussion

While the promise of spectrally diverse fluorophores and reporter strains is exciting for the study of mitochondrial dynamics, the choice of proper FPs or probes is crucial for success in most applications. These probes and live imaging approaches can be used to understand basic biological mechanisms of mitochondrial dynamics, and have already given ground-breaking insight into mitochondrial dynamics and answered some basic questions around mitochondrial biology.

Impediments to the further development of live imaging include: (1) the stability of FPs and probes, (2) the intensity of readily detectable fluorescence that is required for live imaging, (3) the toxicity of many FP fusion proteins when expressed at readily detectable levels and probes that may affect mitochondrial metabolism, and (4) the availability of reagents for directing lineage-specific FP expression.

Advances in live imaging are facilitating the visualization of development at single-cell resolution in living zebrafish embryos (Andersen *et al*. 2010) or *C. elegans* (Cornaglia *et al*. 2015). As reflected in these studies, FPs have immense potential for live imaging applications. We can predict that high-resolution microscopy will give a deeper understanding of mitochondrial dynamics in living mammalian.

Super-resolution microscopy has already made headways in mitochondrial biology (Jakobs and Wurm 2014) but significant limitations remain in live and multi-color applications of the higher resolution approaches of STED, PALM, and STORM. Progress with new fluorophores/fluorescent proteins, especially ones that can utilize longer excitation wavelengths, are expected to move this field forward. Furthermore, development of protocols for quantitative data analysis will greatly enhance the information that can be harvested from STORM/PALM datasets.
